# Anencephalous Births

**Published:** 1847-11

**Authors:** 


*Anencephalous Births.*—Dr. Simpson states that, in his opinion, in anencephalous monsters the malformation arises from intra-uterine disease, viz., from the bursting of the head when hydrocephalic. The brain is opened up and distended by fluid so that it becomes gradually absorbed; at length the enclosing membrane gives way. The two small tubercles, always seen in anencephalous cases lying on the base of the cranium, seem to be nothing else than the remains of the membranes, shrunken up and almost obliterated.—*Ibid.*

